# Expression of heparanase in soft tissue sarcomas of adults

**DOI:** 10.1186/1756-9966-33-39

**Published:** 2014-05-10

**Authors:** Olga Kazarin, Neta Ilan, Inna Naroditzky, Ofer Ben-Itzhak, Israel Vlodavsky, Gil Bar-Sela

**Affiliations:** 1 Division of Oncology, Rambam Health Care Campus, 8 Ha’Aliyah Street, Haifa 35254, Israel; 2 Cancer and Vascular Biology Research Center, The Bruce Rappaport Faculty of Medicine, Technion-Israel Institute of Technology, Haifa, Israel; 3 Pathology Institute, Rambam Health Care Campus, Haifa, Israel

**Keywords:** Expression, Heparanase, Recurrence, Sarcoma, Soft tissue

## Abstract

**Background:**

Heparanase is an endo-β-D-glucuronidase that cleaves heparan sulfate chains of proteoglycans, resulting in the disassembly of the extracellular matrix. Heparanase has a central role in the development of various tumors, and its expression has been associated with increased tumor growth, angiogenesis and metastasis, but there is insufficient information about the function of heparanase in sarcomas.

**Study aims:**

1) To evaluate heparanase levels in adult soft tissue sarcomas (STS); 2) To examine the correlation between heparanase levels and pathological and clinical parameters and treatment outcome.

**Methods:**

Pathological specimens of primary or metastatic STS were subjected to immunohistochemical analysis applying an anti-heparanase antibody. The clinical and the pathological data, together with the data of heparanase levels, were evaluated in a logistic regression model for tumor recurrence and survival.

**Results:**

One hundred and one samples were examined, 55 from primary tumors and 46 from metastatic sites. A high expression of heparanase was observed in 29 (52.7%) and 22 specimens (47.8%), respectively. There was no statistically significant difference between heparanase expressions in the primary vs. metastatic sites of tumors. Moreover, no correlation was observed between heparanase staining and tumor aggressiveness, tumor recurrence or patient survival in various groups of patients.

**Conclusion:**

Expression of heparanase was observed in 50% of the STS, in various histological subtypes. A larger study with homogenous groups of specific sub-types of STS or stages of disease is required to validate over-expression of heparanase as a marker of disease aggressiveness.

## Introduction

Heparanase is an endo-β-glucuronidase that cleaves heparan sulfate (HS) side chains, presumably at sites of low sulfation, releasing saccharide products with appreciable size (4–7 kDa) and biological activity.

“Enzymatic degradation of HS contributes to disassembly of extracellular matrix (ECM) and is therefore involved in fundamental biological phenomena associated with tissue remodelling and cell migration, including inflammation, neo-angiogenesis and metastases formation [1-4]”.

The clinical significance of the enzyme in tumor progression emerged from a systematic evaluation of heparanase expression in primary human tumors. Immunohistochemistry, *in situ* hybridization, RT-PCR and real time-PCR analyses revealed that heparanase is up-regulated in essentially all human carcinomas examined and in some hematological malignancies (i.e. myeloma) [2,5-7]. Notably, increased heparanase levels were most often associated with reduced patient survival post-surgery, increased tumor metastasis and higher microvessel density [2,7,8], thus critically supporting the intimate involvement of heparanase in tumor progression and encouraging the development of heparanase inhibitors as anti-cancer therapeutics [9,10]. Importantly, heparanase up-regulation in human tumors (i.e. head & neck, tongue, hepatocellular, breast and gastric carcinomas) is associated with tumors larger in size [2,8]. Likewise, heparanase over-expression enhanced [11-13], while local delivery of anti-heparanase siRNA inhibited [14] the progression of tumor xenografts, implying that heparanase function is not limited to tumor metastasis but is also engaged in accelerated growth of the primary lesion [12].

While the clinical significance of heparanase in human carcinomas is well documented and anti-heparanase compounds are being tested in clinical trials [15], the role of heparanase in mesenchymal tumors such as sarcoma has not been investigated in detail [16].

Suppressing heparanase levels as a treatment approach was tested using pre-clinical models in various forms of cancer [17-19]. A chemical preparation known as PI-88, which suppresses heparanase activity, was tested in a number of Phase 2 clinical studies and showed some activity when used in the treatment of metastatic melanoma [17], and in conjunction with docetaxel to treat hormone-resistant prostate cancer [18].

The incidence of sarcomas constitutes approximately 1% in adults and up to 12% in children of all malignancies. Approximately 80% have soft tissue origins while the rest originate in the bone. Soft tissue sarcomas (STS) are divided by the World Health Organization (WHO) into more than 50 sub-groups and types. The presence of metastases during the initial diagnosis is uncommon. However, the potential for metastatic disease is elevated according to tumor grade, depth of penetration, and histology [20]. In a break-through laboratory study on animals based on STS models, the experimental drug, heparanase inhibitor SST0001,was administered by subcutaneous injections to tumors with increased heparanase expression, in conjunction with antiangiogenic agents (bevacizumab, sunitinib). The purpose of this treatment was to suppress heparanase activity, resulting in the suppression of growth factors such as VEGF, HGF, and PDGF. The results of this study were positive and complete remission was noted in some of the cases [19].

The primary goal of the current study was to examine the expression of heparanase in soft tissue sarcomas in adults according to common histological sub-types. The secondary goal was to examine the possibility that the over-expression of heparanase serves as a prognostic index in the development of STS metastases.

## Materials and methods

### Sample size

Following approval of the study by the Rambam Health Care Campus Helsinki Committee, 101 biopsies from adult patients diagnosed with STS in the years 2001–2010 and under the care of the Division of Oncology at Rambam Health Care Campus were collected. A number of samples from common types of histology were randomly selected. Data was collected from the clinical follow-up, including demographic and clinical characteristics, stage of disease (TNM) at the time of diagnosis, evidence of recurrence, appearance of outlying metastases, and patient survival.

Patients were excluded if only partial data was available in the medical file, or if it was impossible to prepare enough slides from the pathological block.

Biopsy samples were taken from a primary tumor or from metastases. In 10 cases, the biopsies were taken at different stages of the disease, from a primary tumor and from the metastatic lesion. Biopsies were subjected to immunostaining, applying an antibody (#733) raised against the N-terminal region of heparanase [21], essentially as described [22,23]. Briefly, slides were deparaffinized, rehydrated, and subjected to antigen retrieval by boiling (20 min) in 10 mM citrate buffer, pH 6.0. Following washes with phosphate buffered saline (PBS), slides were incubated with 10% normal goat serum (NGS) in PBS for 60 min to block non-specific binding and incubated (20 h, 4°C) with antibody 733, diluted 1:100 in blocking solution. Slides were extensively washed with PBS containing 0.01% Triton X-100 and incubated with a secondary reagent (Envision) according to manufacturer’s (Dako, Glostrup, Denmark) instructions. Following additional washes, color was developed with AEC reagent (Dako), sections were counterstained with hematoxylin and mounted, as described [21]. Immunostained specimens were examined by a senior pathologist (IN) who was blind to the clinical data of the patients and scored according to the intensity of staining (0: none, +1: weak-moderate; +2: strong). Specimens that were similarly stained with mouse IgG, or by applying the above procedure but lacking the primary antibody, yielded no detectable staining.

### Processing results and statistics

The frequency of over-expression of heparanase based on sub-types of sarcoma and in groups of patients with metastases or with primary cancer was calculated. Using a bivariate logistic regression, a comparison was made between the demographic data, the disease characteristics and the degree of heparanase staining, disease recurrence and survival using the Chi-square test. Confidence Interval (CI) (95%) was calculated according to the sample size and the number of cases with heparanase over-expression.

The level of significance selected to check the various statistical hypotheses in this study was set at p ≤ 0.05. The data was processed using SPSS statistical software, version 18.0 (Chicago, IL).

## Results

**Figure 1:**
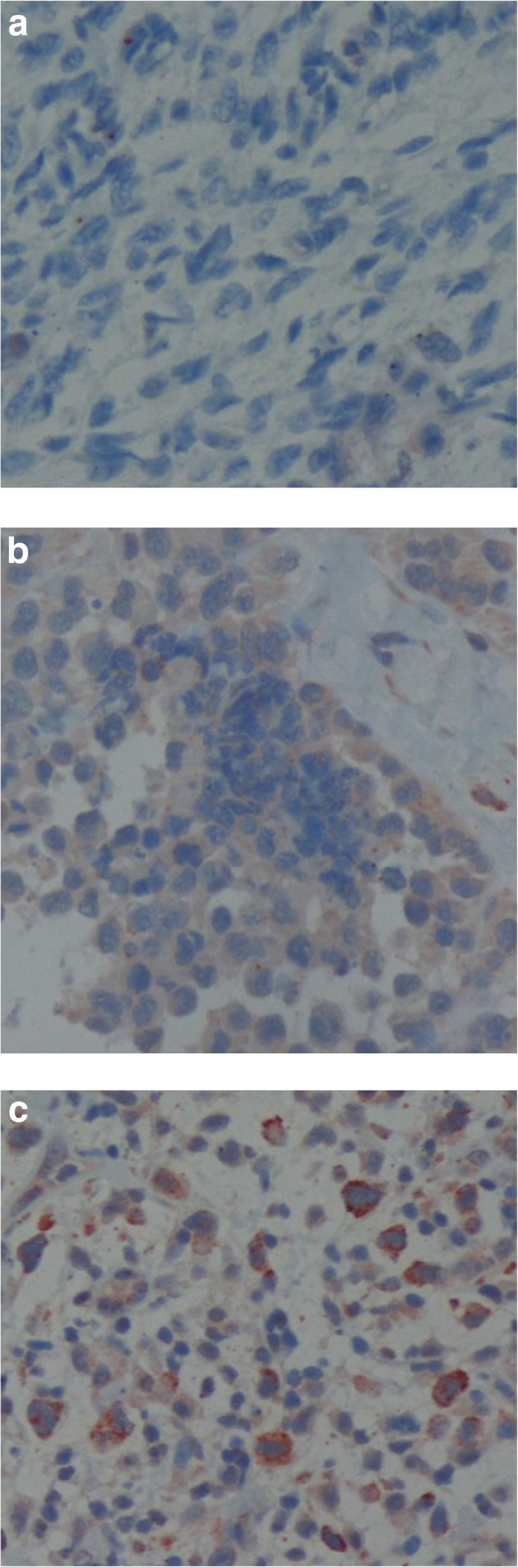
**Examples of heparanase staining in different soft tissue sarcoma types. a:** Negative heparanase staining in leiomyosarcoma, (original magnification × 200). **b:** Weak cytoplasmic heparanase staining in synovial sarcoma, (original magnification × 200). **c:** Strong cytoplasmic heparanase staining in malignant fibrous histiocytoma, (original magnification × 200).

One hundred and one patients were included in the study. The main patient demographic and clinical characteristics are summarized in Table 1. Fifty-eight were male. Median age at diagnosis was 63 years; 59 (58.6%) patients were over the age of 60. Thirty percent of the patients had malignant fibrous histiocytoma (MFH) and 22% of the patients were diagnosed with a given sarcoma with no defined sub-type histology (NOS). Two-thirds (66%) of the patients had high grade sarcomas. Nearly 20% of the patients had metastatic disease at the time of diagnosis. All 101 histological specimens of STS were stained for heparanase as described above, 55 from primary tumors and 46 from metastatic sites. A high expression of heparanase was seen in 29 (52.7%) and 22 specimens (47.8%), respectively. Figure 1(a-c) shows different samples of STS stained for heparanase, with negative, low and positive heparanase expression accordingly.

**Table 1:** Demographic and clinical data for 101 patients related to over-expression of heparanase based on IHC staining

**Characteristic**		**No. of patients out of entire group**	**No. of patients with over-expressed heparanase, according to sub-groups (%)**	**P value**
**Age**	<40	21	6 (28.5%)	0.65
	40-59	21	11 (52.4%)	
	60-69	30	16 (53.3%)	
	>70	29	12 (43.3%)	
**Gender**	Male	58	25 (43.1%)	0.88
	Female	43	20 (46.5%)	
**Pathological type**	Malignant fibrous histiocytoma	30	12 (40%)	0.87
	Liposarcoma	16	8 (50%)	
	Leiomyosarcoma	13	6 (46.1%)	
	Angiosarcoma	4	1 (25%)	
	Chondrosarcoma	7	5 (71.4%)	
	Sinovial sarcoma	9	4 (44.4%)	
	NOS	22	9 (40.9%)	
**Grade**	Low	28	12 (42.8%)	0.44
	Intermediate	6	2 (33.3%)	
	High	67	31 (46.2%)	
**Stage**	I	29	13 (44.8%)	0.55
	II	7	1 (14.3%)	
	III	46	20 (43.4%)	
	IV	19	11 (57.9%)	
**Total**		**101**	**51 (50.5%)**	

Table 1 summarizes the correlation between over-expression of heparanase in the pathological samples and the clinical and pathological characteristics of the patients. The staining was graded according to the strength of the color and its perimeter, as detailed in Methods and Materials. More than 95% of the pathological samples stained for heparanase in over 50% of the cells; therefore, it was not possible to analyze the data based on the extent of the staining. In general, heparanase over-expression was seen in nearly 50% of the samples and in all sub-groups of histological sub-types, pathological grade or stage of disease.

Estimation of the correlation between the color strength of the stain for heparanase and the risk of the disease recurring was performed on 55 patients with biopsy samples taken from a primary tumor following radical surgery to remove the tumor.

During the follow-up period over at least five years from the time of the surgery, the disease recurred in 50% of the patients. In half the patients whose disease recurred during the clinical follow-up period, strong color staining for heparanase was observed, although the same was also observed in 12 samples from 29 patients whose disease did not recur. Accordingly, the sensitivity and specificity of the strong color staining for heparanase as a predictor for the recurrence of the disease are 0.50 and 0.59, respectively.

Table 2 summarizes the risk for disease recurrence according to demographic and histologic parameters for each group. A statistically significant risk for disease recurrence was found only to grade and stage of the disease.

**Table 2:** Disease recurrence according to demographic and histologic parameters, in 55 patients

**Characteristic**		**No. of patients out of entire group (%)**	**No. of patients with recurrent disease (% of each sub group)**	**Disease recurrence p value**
**Age**	<40	13 (24%)	5 (38.5%)	0.73
	40-59	9 (16%)	4 (44.4%)	
	60-69	14 (25%)	8 (57.1%)	
	>70	19 (35%)	10 (52.6%)	
**Gender**	Male	33 (60%)	16 (48.5%)	0.44
	Female	22 (40%)	12 (54.2%)	
**Pathological type**	Malignant fibrous histiocytoma	19 (35%)	12 (66.7%)	0.67
	Liposarcoma	8 (15%)	3 (37.5%)	
	Leiomyosarcoma	6 (11%)	4 (66.6%)	
	Angiosarcoma	2 (4%)	0	
	Chondrosarcoma	5(8%)	1 (20%)	
	Synovial sarcoma	4 (7%)	3 (75%)	
	NOS	11 (20%)	5 (45.5%)	
**Grade**	Low	15 (27%)	0	0.01>
	Intermediate	3 (5%)	1 (33%)	
	High	37 (67%)	27 (73%)	
**Stage**	I	18 (33%)	1 (5.5%)	0.01>
	II	4 (7%)	3 (75%)	
	III	33 (60%)	24 (73%)	
**Level of heparanase expression**	No staining	5 (9%)	3 (60%)	
	Weak staining	18 (33%)	10 (55%)	0.77
	Strong staining	32 (58%)	15 (47%)	

In 10 cases, biopsy specimens from metastases were compared to the surgical specimens of the primary tumors. In 8/10 cases, no difference in the level of staining was observed. The sample was too small for any statistical analysis (Table 3).

**Table 3:** Level of heparanase staining in samples from the primary tumor and metastases of the same patients

**Depth of stain color for heparanase**	**Sample from primary tumor**	**Sample from metastases**
**Strong (2)**	7	5
**Weak (0–1)**	3	5
**Total**	**10**	**10**

## Discussion

The primary endpoint of the current study was to check the expression of heparanase using immunohistochemistry staining of tumor samples taken from soft tissue sarcomas in adults. A limited number of studies have checked heparanase levels in different sarcoma types, including a study by Shafat et al. [16], which examined the level of heparanase in pathological samples taken from children with Ewing’s sarcoma. Heparanase levels were evaluated using immunohistochemistry of 69 pathological samples utilizing methodology similar to that applied in this study. Over-expression of heparanase was seen in 51% of the cases. In another study, Masola et al. examined the expression of heparanase in 15 pathological samples and in the blood of children with rhabdomyosarcoma [24]. While pathological specimens were stained positive for heparanse, the level of the enzyme in the blood was similar to healthy controls. The current study is the first attempt to evaluate the level of heparanase over-expression in sarcoma that frequently occurs in adults, showing a similar percentage of over-expression as in children’s sarcoma subtypes.

A number of studies have found high levels of heparanase in tumor cells in comparison to normal and pre-cancerous cells [25,26]. For example, Maxhimer et al. reported a high prevalence of heparanase expression in breast tumor tissue at advanced stage (53%), in comparison to tumors at an early stage of the disease (23%) and in healthy breast tissue (0%) [27].

A study by Friedmann et al. [22] examined the level of heparanase in the mucous membrane of the colon and colon polyps and neoplasm, using an mRNA probe directed against heparanase (*in situ* hybridization) and immunostaining. Heparanase expression increased when the level of cellular differentiation was lower and the dysplasia was higher, while there was almost no heparanase expression in normal cells. High expression of heparanase was found in primary colon cancer as well as in colon cancer metastases to the lungs, liver, and lymph nodes. In the sarcomas, the tissue of mesenchymal origin where the tumor forms is usually not defined. It is therefore not possible to document the heparanase level during the developmental stages of the tumor. As opposed to breast carcinoma but similar to colon carcinoma, the current study found a similar rates of heparanase over-expression in primary tumors and metastases.

Most of the studies that addressed the question of heparanase expression were carried out on epithelial tumors. A large number of these studies found a direct connection between the enzyme level in the tumor and the aggressiveness of the disease, implying that the heparanase level could function as a prognostic predictor [25-27]. The secondary end-point of the current study attempts to test the prognostic significance of heparanase expression after ascertaining that the prognostic factors known from the literature (grade and stage) are indeed repeated in this study. No correlation was found between heparanase levels and prognosis. It is possible that, due to the high level of heterogeneity of the various histological types of sarcoma, a much larger sample group would be required to reveal the role of heparanase as a prognostic factor in sarcomas. In contrast to the current study, the study by Shafat et al. [16] found a correlation between heparanase level and poor prognostic factors (tumor size and patient age at time of diagnosis) in Ewing’s sarcoma. It is noteworthy that there is a significant difference between the course of the disease, prognosis, and treatment for patients with STS in adults and common sarcomas in children [28].

## Conclusions

Heparanase expression was increased in more than 50% of the STS cases. We were unable to find a correlation between heparanase staining intensity and recurrence of the disease. In light of the development of heparanase inhibitors as novel treatment options, it is important to carry out further studies, which should include larger patient groups with specific sub-type sarcomas, in order to better delineate the significance of heparanase in STS.

## Competing interests

The authors declare that they have no competing interests.

## Authors’ contributions

OK carried out the histological staining and collected the clinical data. NI was responsible for the heparanase laboratory, including the staining, and helped to draft the manuscript. IN and OBI deciphered the stained samples. IV participated in the design of the study and helped to draft the manuscript. GB analyzed the pathological and clinical data, made the statistical analysis, and wrote the manuscript. All authors read and approved the final manuscript.
